# Glances at Foreign Asylums

**Published:** 1890-06-14

**Authors:** 


### Glances at Foreign Asylums.

[Annual Reports, Pamphlets, &c., should be addressed to The Editor, The Lodge, Porchester Square, London, W.]

### HOSPITAL FOR THE INSANE, PARAMATTA, NEW SOUTH WALES.

This asylum contains nearly 1,000 beds, and during the year it has increased its population by 20. It is much to be desired that the Colonial authorities will set their faces stubbornly against any further enlargement of these institutions, otherwise they will find themselves saddled with unwieldy hospitals for the insane, just as we are in England, which are not suitable for their purposes, and cannot be productive of good.

It is pleasing to note that the recovery rate is rising and the death rate falling. A leaf has been taken out of our English book, for we find that epileptics and suicidal cases are collected into a separate ward at night and placed under continuous supervision. Dr. Godson does not tell us what his experience of the innovation has been. It would seem that restraint was used in the cases of four women for special reasons. Dr. Manning says that "care should be taken so as to keep the number under treatment in this way as small as possible."

### EASTERN MICHIGAN ASYLUM AT PONTIAC.

Here we find a comparatively new asylum opened in 1878. At the end of last year the number of patients was 775. During the year 251 cases were admitted; 61 were discharged recovered, and 56 died.

Dr. Hurd's report is a very exhaustive one. Among other things, he gives details of many of the re-admissions and assigns probable causes for the return of some of them. We are favoured with a very interesting synopsis of the history of a typical case of the drunken "ne'er do well." After being in a variety of gaols and asylums, he turned up at Michigan, where he was at the date of Dr. Hurd's report. "He is now free from any symptoms of mental disease, and cannot be retained much longer. What shall be done with him? If he does not indulge in alcoholics he is able to earn good wages, and is self-supporting and respectable. It is, however, absolutely certain that he will indulge these vicious tastes, and suffer renewed attacks of insanity." We know this type well, and we have many a time asked, "What is to be done with him?"

### CALLAN PARK HOSPITAL FOR THE INSANE, NEW SOUTH WALES.

In this asylum the average number resident was 702. The recoveries reached the creditable percentage of 46·57 on the admissions, and the deaths stood at 8·26 on the average number resident. The asylum would seem to be under good management. The airing courts have been laid out in flower-beds, and the wards are well supplied with pet animals and such things as are likely to interest the patients. Dr. Blaxland says that even the most irritable patients rarely interfere with any of these.

On looking at the table showing the causes of the insanity, we find that drink is set down as the most potent factor, not fewer than 47 out of the 279 admissions being due to that scourge. Hereditary influence only accounts for 19; but this figure is certainly below the mark, probably owing to the difficulty always found in getting reliable information on that point.

Of the 57 deaths, 7 were owing to consumption.

Dr. Manning says he has "to express the greatest satisfaction with the general condition of the male division," and that "the female division is rapidly improving."

